# Non-carcinogenic and cumulative risk assessment of exposure of kitchen workers in restaurants and local residents in the vicinity of polycyclic aromatic hydrocarbons

**DOI:** 10.1038/s41598-023-33193-0

**Published:** 2023-04-24

**Authors:** Narges Shamsedini, Mansooreh Dehghani, Mohammad Reza Samaei, Majid Nozari, Shayan Bahrany, Zeynab Tabatabaei, Aboolfazl Azhdarpoor, Mohammad Hoseini, Mohammad Fararoei, Sareh Roosta

**Affiliations:** 1 Department of Environmental Health Engineering, School of Health, Student Research Committee, Shiraz University of Medical Sciences, Shiraz, Iran; 2 Fars Water and Wastewater Company, Shiraz, Iran; 3 Research Center for Health Sciences, Institute of Health, Department of Environmental Health Engineering, School of Health, Shiraz University of Medical Sciences, Shiraz, Iran; 4 Department of Environmental Health Engineering, School of Public Health, Kerman University of Medical Sciences, Kerman, Iran; 5 Otolaryngology Research Center, Shiraz University of Medical Sciences, Shiraz, Iran

**Keywords:** Environmental sciences, Biomarkers, Health occupations, Risk factors

## Abstract

Polycyclic aromatic hydrocarbons (PAH_s_) are often formed when organic substances do not burn completely. This study evaluates the non-carcinogenic and cumulative risks associated with PAH_s_ levels by testing blood and urine samples in kitchen workers and residents near restaurants in Shiraz, Iran. Metabolites of PAH in the urine samples as well as clinical parameters in the blood samples were measured. The non-carcinogenic and cumulative risk assessments from exposure of the study groups to PAH metabolites were also evaluated. The highest average concentrations of PAH metabolites were related to kitchen workers (2126.7 ng/g creatinine (ng/g cr)). The metabolites of 1-Hydroxypyrene (1-OHP) and 9-Phenanthrene (9-OHPhe) had the highest and lowest mean concentrations, respectively. A direct correlation was observed between the levels of PAH metabolites with malondialdehyde (MDA) and total antioxidation capacity (TAC) levels (p < 0.05). Hazard Index (HI_i_) was obtained less than one (HI_i_ < 1), indicating low-risk negative health impacts on the target groups. Nevertheless, conducting more studies to determine the health status of these people is quite evident.

## Introduction

The incomplete combustion of organic waste results in a unique type of environmental pollutant known as PAHs^1,2^. These contaminants stay in the ecosystem and build up in the food supply^3^. People may be exposed to these pollutants from different sources and routes^1^. The most common routes to get exposure to PAHs are inhalation, smoking, food, soil, air, and skin contact^4^.

Endocrine system disruption is caused by PAHs, which are toxic, cancer-causing, and mutagenic substances. The International Agency for Research on Cancer (IARC) has classified benzo[a]pyrene (BaP) as a carcinogen to humans (group 1)^4^. The US Environmental Protection Agency (USEPA) has likewise classified them as priority pollutants. PAH_s_ can generate reactive oxygen species (ROS). Furthermore, a strong positive association between exposure to PAH_s_ and indices of oxidative stress has been reported in several studies^5,6^. Free radicals at the cell membrane cause oxidative stress, which damages the cell membrane and results in cell malfunction. In actuality, PAH metabolites damage membranes and raise levels of aldehydes such as MDA^7^.

When food is fried, stir-fried, or grilled in cooking oil at high temperatures, cooking oil fumes (COFs) like polycyclic aromatic hydrocarbons (PAHs) are produced and released into the environment^8^. The inhaling, eating, and even skin absorption of unprotected restaurant staff exposes them to dust and gaseous emissions containing PAH_s_. Restaurant employees and those who live in or close to that region are at risk of health problems due to such PAH_s_ emissions. Since several PAH metabolites are known to cause cancer, individuals' health may suffer due to these contaminants^9,10^. The presence of PAH metabolites as biomarkers, such as 1-hydroxynaphthalene (1-OHNap), 2-hydroxynaphthalene (2-OHNap), 2-hydroxyfluorine (2-OHFlu), and 9-OHPhe in the urine is one sign that restaurant workers have been exposed to COF^1,5,11–13^.

Too far, many studies have been carried out to assess PAH_s_ concentrations in various places, including bus terminals, workplaces, and dining establishments^14–18^. According to certain studies, those who work in restaurants are more likely to develop certain malignancies^10,17^. For example, research on the concentration of PAH metabolites in the urine of Indian kitchen employees revealed that it was more significant in the workers than in the control group^17^. In another study, researchers found PAH_s_ and benzo[a]pyrene concentrations at Chinese restaurants were 20.99 ± 13.67 and 1.82 ± 2.24 μg/m^3^, respectively. Furthermore, they stated that based on an incremental lifetime cancer risk (ILCR) of less than 10^–6^, the maximum allowable exhaust stack exposure duration for Chinese citizens was 5–19 h/month^−1^^19^. Most of the information currently accessible on the environmental harm caused by PAH_s_ emissions derives from China and India. No data on PAH_s_ exposure related to kitchen employees have been published from Shiraz, Iran, which has many restaurants. In this study, we investigate PAH metabolites in kitchen employees and persons residing near restaurants in Shiraz, Iran, as well as controls (who were not exposed to restaurant emissions). In this regard, metabolites of PAH (1-OHNap, 2-OHNap, 2-OHFlu, 1-OHP, 9-OHPhe, MDA, and TAC) were measured in urine samples. Also, clinical parameters such as White Blood Cells (WBC), Red Blood Cells (RBC), C-Reactive Protein (CRP), etc., were measured in the blood samples of the studied groups. Finally, non-carcinogenic and cumulative risk assessment studies were conducted.

## Materials and methods

### Location, statistical population, and study inclusion criteria

This cross-sectional research was done on kitchen employees and those living near restaurants in Shiraz, Iran. The sample size was determined using data from prior research to discover the biggest difference between the two exposure groups (type I and type II) and the control group. The statistical population of the study included 144 people. These included 57 people working in restaurant kitchens, 57 people living near restaurants, and 30 people as a control group (people who do not work or live near the restaurant). We also matched people from various groups based on frequency and age. Inclusion criteria for the study included living in the city 3 days before the test, not using acetaminophen, adult cold medicine, and nutritional supplements for at least 3 days before the sampling date, and so on.

### The steps of collecting questionnaire information and taking blood and urine samples from people

All individuals signed written informed consent forms before sampling. A questionnaire with 55 questions was given to individuals to gather information on their sociodemographic traits, activity level, dietary intake of PAH_s_, and smoking experience. The questionnaire included age, lifestyle, residence features, dietary patterns, environmental tobacco smoke (ETS), and time spent outside. Information from the individuals was requested after 72 h. Face-to-face interviews were conducted with each participant.

We also performed anthropometric measurements after the interview, including weight, height, and blood pressure. The National Health and Nutrition Examination Survey (NHANES) methodology was used for measuring height and weight^20^. After a ten-minute break, blood pressure was checked on the right hand using a blood pressure monitor (OMRON, M2 Japan).

The samples (blood and urine) were collected in the morning. Samples were immediately transported to a laboratory at 0–4 °C, where they were maintained at − 20 °C until analysis. An automated biochemistry analyzer (Poch-100i, Sysmex, Kobe, Japan) also evaluated high-density lipoprotein cholesterol (HDL-C), low-density lipoprotein cholesterol (LDL-C), total cholesterol (TC), triglycerides (TG), and fasting blood sugar (FBS). The liver function enzymes (sGpt and sGot) were also measured along with TAC, MDA, CRP, and creatinine. Additionally, this study was approved by the Ethics Committee of Shiraz University of Medical Sciences (IR.SUMS.REC.1399.703), and all methods were performed in accordance with the relevant guidelines and regulations^21^.

### Urine sample preparation and analysis

We combined 6 mL of the urine sample with 12 mL of sodium acetate (C_2_H_3_NaO_2_) buffer (0.1 M, pH 5) to determine the PAH metabolites (1-OHNap, 2-OHNap, 2-OHFlu, 1-OHP, and 9-OHPhe). 80 μL of β-glucuronidase/arylsulfatase was added to the mixture and hydrolyzed for five hours in a water bath at 37 °C. To separate the supernatant, the mixture was then shaken for 15 min at 3000 rpm. A Gas Chromatography (GC) vial was filled with the supernatant. In order to quantify the PAH metabolites, 3 mL of this supernatant was injected into the GC–MS apparatus (Agilent, HP5-MS capillary column, 7890, USA)^22,23^. To adjust the concentrations of PAH metabolites, the creatinine (ng/g cr) was determined according to Jaffe’s spectrophotometric technique at the ultraviolet (500 nm) absorption spectrum^4^.

### Determination of the estimated daily intake (EDI_i_), hazard quotient (HQ_i_), and hazard index (HI_i_)

Equation (1) was applied to the calculation of EDI_i_^24^.

1 $${\mathrm{EDI}}_{\mathrm{i}}=\frac{\mathrm{ CU}\times {V}_{24h}\times \mathrm{MW}}{\mathrm{FUE}\times \mathrm{BW}}$$

In Eq. (1), EDI_i_ is equal to the estimated daily intake of PAH_s_ (nanogram per kilogram body weight per day), CU is the molar concentration of PAH metabolites in urine (nmol/L for EDI_i_), and V_24h_ is the total volume of urine excreted during 24 h for adults (1.6 L per day).The molecular weight of the parent PAH_s_ constituents (Nap, Flu, Phe, and Pyr of 128.17, 166.22, 178.23, and 202.26 g/mol, respectively) are expressed by MW. FUE refers to the amount of urine PAH metabolites excreted as a fraction of oral intake (Nap, Flu, Phe, and Pyr of 1, 0.78, 0.09, and 0.08, respectively), and BW is body weight^25^.

The HQ_i_ was used to evaluate the non-carcinogenic risk (Eq. 2)^4,24^:

2 $${\mathrm{HQ}}_{i}=\frac{EDIi}{RFDi}$$

In Eq. (2), HQ_i_ and RFD_i_ are the hazard quotient and reference dose for oral exposure (Nap = 20, Flu = 40, Phe = 30, and Pyr = 30 µg/kg-BW day), respectively. Furthermore, the HI_i_ was computed to assess the cumulative risk of exposure to dietary PAHs (Eq. 3)^4,24^:

3 $${\mathrm{HI}}_{\mathrm{i}}=\sum {\mathrm{HQ}}_{\mathrm{i}}$$

Low-risk adverse health impacts on the target population were reflected by HI_PAHs_ < 1.

### Statistical analysis

SPSS 21 software was used to perform all analyses. The Shapiro–Wilk normality analysis was used to check the data distribution. The Mann–Whitney U analysis was used to compare the measured variables' values between various groups. These exposure categories included living near restaurants, workshops, or factories, being near busy roads, car repairs, bus stops, passive smoking, the type of heating appliance used inside the home, and kitchen hoods. The significant correlation between the two variables was also determined using the Spearman correlation. Multivariate linear regression analysis was used to evaluate the relationship between PAH metabolites levels and independent factors that may affect their concentration in urine.

### Ethical approval

The Shiraz Medical University's ethics committee in Shiraz, Iran, approved this work (ethics code: IR.SUMS.REC.1399.703).

### Consent to participate

I consent to participate in the research project and the following has been explained to me: (1) the research may not be of direct benefit to me; (2) my participation is completely voluntary; (3) my right to withdraw from the study at any time without any implications to me; (4) the risks including any possible inconvenience, discomfort or harm as a consequence of my participation in the research project; (5) the steps that have been taken to minimize any possible risks; (6) public liability insurance arrangements; (7) what I am expected and required to do; (8) whom I should contact for any complaints with the research or the conduct of the research; (9) I am able to request a copy of the research findings and reports; (10) security and confidentiality of my personal information.

## Results and discussion

### Characteristics of the participants

Table 1 presents the demographics of the participants (control group (n = 30), kitchen workers (n = 57), and individuals who live close to restaurants (n = 57)). There were 49 (87.5%) males and 8 (12.5%) females among the participants (kitchen workers and people living near restaurants). Among the control group, 25 (83.33%) were male, while 5 (16.67%) were female. The average age of kitchen workers, people living near the restaurant, and the control group was 33.6, 33.9, and 33.41 years, respectively. The participant's body weight index (BMI) ranged from 24.7 to 25.46 kg/m^2^. According to the findings, 37 participants were exposed to passive smoking for 48 h before sampling. The most common transportation by volunteers was by private car and taxi. More than 70% of each group used a kitchen hood. Over half of the participants lived close to sources of PAH_s_ emissions, including bus terminals, parking lots, and traffic areas.

**Table 1:** Specifications of the studied groups in terms of socio-demographics.

	Kitchen workers	People living near restaurants	Control group
Sex			
Male (N, %)	49, 87.5	49, 87.5	25, 83.33
Female (N, %)	8, 12.5	8, 12.5	5, 16.67
Age (mean, year)	33.6	33.9	33.41
Height (mean, cm)	174.3	172.4	172.7
Weight (mean, kg)	74.8	75.35	74.5
Body mass index (kg/m^2^)	24.7	25.46	25.1
Second-hand smoke exposure within the past 48 h (N, %)	14, 24.4	18, 31.5	5, 16
Traffic status of the living place (low, moderate or high, %)	66.6/ 31.1/2.2	41.4/ 56.1/2.43	36/56/8
Using a hood while cooking	75	80	70.3

### Distribution of PAH metabolites levels in urine

**Figure 1:**
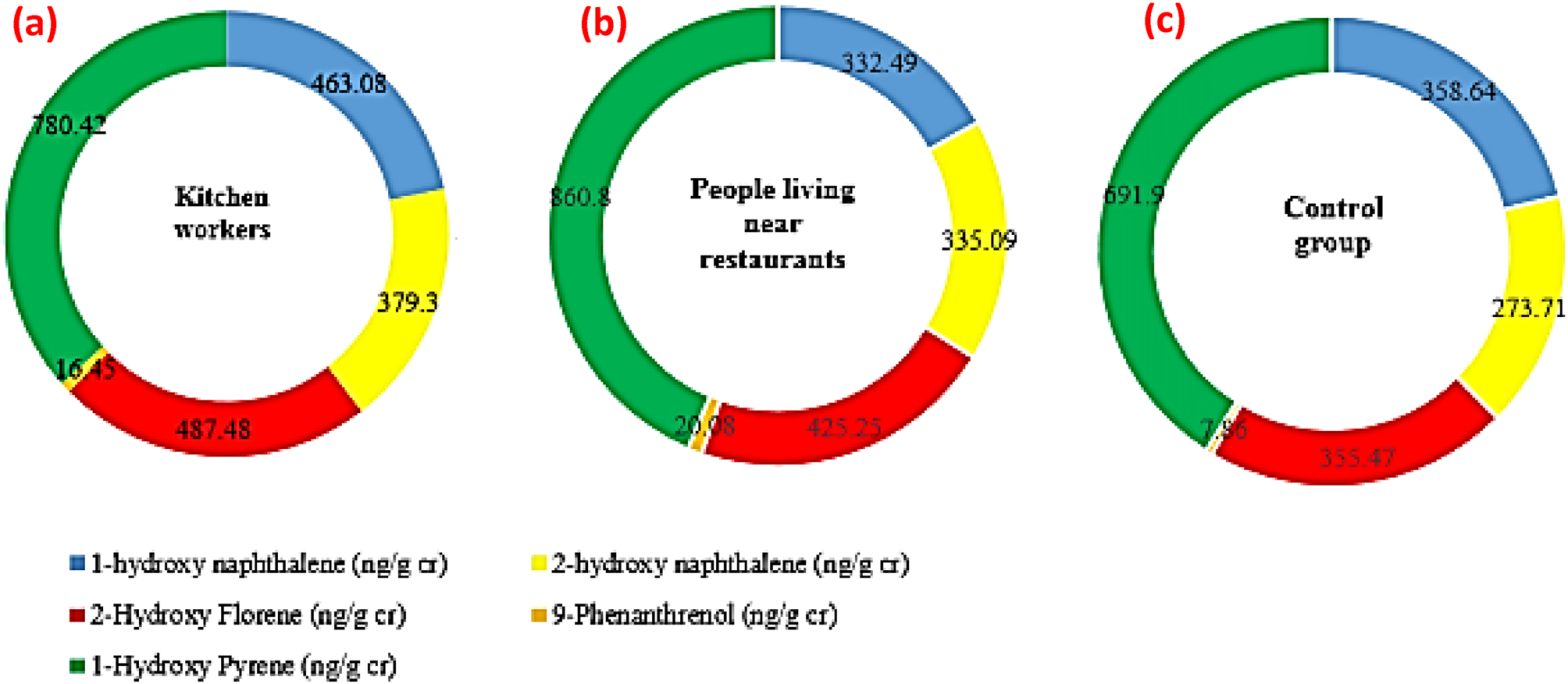
Mean levels of OH-PAH metabolites in the studied group’s urine samples (ng/g cr) ((**a**) kitchen workers, (**b**) people living near restaurants, and (**c**) the control group).

Exposure to PAH_s_ is a significant concern because of its effects on human health. The mean levels of PAH metabolites in the urine samples of the study groups are displayed in Fig. 1 and other details are given in Table S1 (Supplementary data). The highest and lowest mean levels were related to 1-OHP and 9-OHPhe, respectively, in the three study groups. Similar findings were also achieved in research on kitchen workers in India^13^. Another research found that 1-OHP was more prominent than other PAH metabolites^26^. Further, the highest mean level of PAH metabolites measured in a study of PAHs biomonitoring adults in a Middle Eastern region was 1-OHP^5^, which is consistent with our results. 1-OHP is rapidly excreted through the urine, which could explain its high urinary level^5^.

Kitchen workers, people who live near restaurants, and the control group had a total average concentration of PAH metabolites (ΣOH-PAH_s_) of 2126.7, 1973.7, and 1687.61 ng/g cr, respectively (Fig. 1). Kitchen workers and those living near restaurants had higher ΣOH-PAH_s_ than the control group, but this difference was only significant between the workers and control groups (p value < 0.05). A study in India confirmed this finding, revealing that kitchen workers had higher ΣOH-PAH_s_ than the control group^27^. The ΣOH-PAHs in the urine of Shiraz citizens were 1988.1 ng/g cr^5^, which was lower than that found among the people who worked in restaurant kitchens. This seems logical because these workers are more exposed to PAH_s_ due to being involved in the frying processes. It was also found in another study on school children in Shiraz that the ΣOH-PAHs (1460 ng/g cr) were lower than those in our study^16^. Differences in age and time spent at work and home might explain the discrepancy between the results.

Based on our findings, a significant difference in 9-OHPhe level was seen between people residing near restaurants and the control group (p = 0.017). According to a study conducted by Siddique, a significant concentration of phenanthrene was formed in all samples after cooking (frying)^28^, so it could be a logical reason for a higher 9-OHPhe level in samples of people living near restaurants (20.08 ng/g cr) than in the control group (7.86 ng/g cr). This result is similar to that reported by Wang et al.^29^. Nevertheless, there was no significant difference in other urinary PAH metabolites concentration among the three groups of our study (p > 0.05).

### Clinical parameters levels in the studied subjects

Descriptive statistics for the clinical parameters of the participants are presented in Table 2. In this study, no significant difference was found between the parameters measured in the different groups (p > 0.05). However, the results of the study by Singh et al.^13^ in India report that continuous exposure to heat in kitchens can impair the kidney function of kitchen workers. Furthermore, in a study of residents of Shiraz, a significant difference in red blood cell (RBC) count and TG level was found between male and female participants^5^. This result was not observed in our study; it is probably due to the fact that in the current study, most of the participants were male.

**Table 2:** A comparison of the levels of the clinical parameters in the blood samples from the different study groups.

	Kitchen workers			People living near restaurants			Control group			p value
	Mean ± SD	Min	Max	Mean ± SD	Min	Max	Mean ± SD	Min	Max	
WBC (10^3^/mic L)	6.87 ± 1.22	3.9	9.6	6.79 ± 1.68	3.7	11.4	6.52 ± 1.27	3.8	8.9	> 0.05
RBC (10^6^/mic L)	5.06 ± 0.55	3.7	6.21	5.11 ± 0.47	4.2	6.4	5.09 ± 0.6	3.59	7	> 0.05
Hb (g/dL)*	15.21 ± 1.87	10	20.5	15.41 ± 1.15	11.8	17.4	15.56 ± 1.44	11.8	17.8	> 0.05
RDW**	13.02 ± 1.03	11.3	16.1	12.87 ± 1	11.5	17	12.83 ± 0.58	11.8	14.5	> 0.05
FBS (mg/dL)	80.19 ± 14.3	62	156	80.87 ± 9.8	61	108	81 ± 10.04	62	108	> 0.05
TG (mg/dL)	116.67 ± 57.56	33	269	140.22 ± 87.56	44	490	128.53 ± 61.03	62	250	> 0.05
Total cholesterol (mg/dL)	173.16 ± 35.8	110	271	170.42 ± 35.68	79	252	180.26 ± 35.87	116	265	> 0.05
HDL-C (mg/dL)	45.37 ± 14.64	34	138	42.05 ± 6.4	30	56	45.36 ± 9.79	35	85	> 0.05
LDL-C (mg/dL)	102.63 ± 28.31	26	178	99.1 ± 29.98	32	179	111.06 ± 28.88	61	176	> 0.05
sGot (IU/L )	17.62 ± 8.12	9	47	17.67 ± 7.76	39	7.76	18.2 ± 8.7	6	37	> 0.05
sGpt (IU/L )	8.18 ± 7.06	2	48	7.56 ± 5.85	30	1	8.6 ± 96	1	40	> 0.05

*Hb (hemoglobin), **RDW (red blood cell distribution with).

### Measure of the concentrations of MDA (µm/mM cr) and TAC (mM/mM cr) in urine samples, and CRP (mg/L) in blood samples

**Figure 2:**
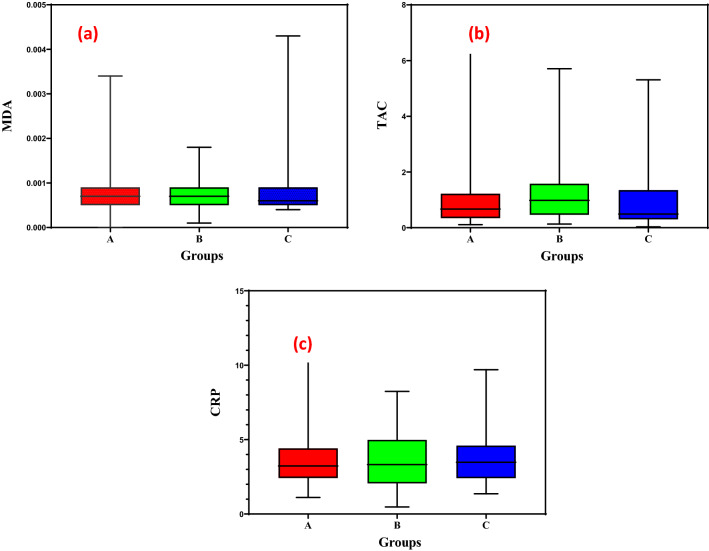
A measure of the average concentrations of (**a**) MDA (µm/mM cr) and (**b**) TAC (mM/mM cr) in urine samples, and (**c**) CRP (mg/L) in blood samples (A: kitchen workers, B: individuals living near restaurants, and C: control group).

Figure 2 shows a measurement of mean concentrations of MDA (µm/mM cr) and TAC (mM/mM cr) in urine samples and CRP (mg/L) in blood samples from study groups.

According to the test results, no significant difference was observed between the three groups in the concentrations of MDA, TAC, and CRP (p value > 0.05). The mean mass concentration of MDA was (0.00082, 0.00075, and 0.00082), TAC (1.21, 1.4, and 1.01), and CRP levels (3.67, 3.65, and 3.65) in A, B, and C, respectively (Fig. 2). Pan et al.^8^ found in their study that kitchen staff (369 µmol/mol cr) had significantly higher urinary MDA levels than service staff (267.2 µmol/mol cr), which contradicts the current study.

Spearman's rank correlation coefficient was used to examine the association between measured concentrations in the study groups. A significant and positive linear relationship between the concentration of red blood cells in blood samples and TAC (CC = 0.33, p = 0.00), CRP (CC = 0.19, p = 0.02), and MDA (CC = 0.17, p = 0.04) was observed. In other words, with increasing RBC concentration, the concentration of TAC, CRP and, MDA increases linearly and this correlation is higher in TAC due to the higher correlation coefficient. Furthermore, RBCs in one study had a direct linear proportional relationship with MDA^30^, which is consistent with our findings.

Moreover, there was a significant negative linear correlation between TAC concentrations and MDA (CC = − 0.22, p = 0.01). This means that as TAC concentration rises, MDA concentration falls linearly and vice versa. In a biomonitoring study, MDA and TAC levels were significantly higher and lower, respectively, in men with idiopathic infertility than in fertile men^31^ which, like the present study, showed a relationship inverse between the TAC and MDA parameters.

### The correlations between PAH metabolites (ng/g cr) and MDA, TAC, and CRP levels

Table 3 shows the correlations between PAH metabolites (ng/g cr) and MDA (mM/mM cr), TAC (mM/mM cr), and CRP (mg/L) in the groups studied.

**Table 3:** Correlations between PAH_s_ metabolites concentration (ng/g cr) and concentrations of MDA (mM/mM cr), TAC (mM/mM cr) and CRP (mg/L) in the study groups.

Groups		A: Kitchen workers			B: People living near restaurants			C: Control group		
PAHs metabolites		CRP	MDA	TAC	CRP	MDA	TAC	CRP	MDA	TAC
1-OHNap	Spearman correlation C	0.081	0.612**	− 0.263*	− 0.081	0.572**	− 0.428**	− 0.132	0.206	− 0.760**
	p value	0.551	< 0.001	0.048	0.552	0.000	0.001	0.496	0.275	0.000
2-OHNap	Spearman correlation C	− 0.047	0.597**	− 0.447**	− 0.175	0.353**	− 0.292*	− 0.083	0.171	− 0.539**
	p value	0.727	0.000	0.000	0.196	0.007	0.028	0.670	0.366	0.002
2-OHFlu	Spearman correlation C	0.065	0.815**	− 0.606**	− 0.071	0.494**	− 0.321*	− 0.146	0.214	− 0.835**
	p value	0.633	0.000	0.000	0.603	0.000	0.015	0.450	0.257	0.000
9-OHPhe	Spearman correlation C	0.061	0.510**	− 0.174	0.102	0.482**	− 0.356**	− 0.046	0.052	− 0.078
	p value	0.650	0.000	0.197	0.457	0.000	0.007	0.815	0.784	0.682
1-OHP	Spearman correlation C	− 0.021	0.597**	− 0.315*	− 0.095	0.385**	− 0.244	− 0.329	0.099	− 0.211
	p value	0.876	0.000	0.017	0.488	0.003	0.067	0.081	0.604	0.263
ΣOH-PAHs	Spearman correlation C	0.022	0.785**	− 0.464**	− 0.121	0.530**	− 0.365**	− 0.261	0.069	− 0.568**
	p value	0.873	0.000	0.000	0.372	0.000	0.005	0.172	0.718	0.001

The results indicated that there was a significant correlation between TAC levels and all measured metabolites of PAH, except for 9-OHPhe in the A and C groups, as well as 1-OHP in the B and C groups. The TAC assay was been applied to determine the antioxidant capacity of some heterogeneous compounds with antioxidant activity in body fluids and thus can also help in assessing the overall antioxidant status. Other research has found that increased exposure to PAH compounds increases oxidative stress and decreases TAC, increasing lung cancer susceptibility^32^.

Furthermore, according to the results shown in Table 3, it can be seen that as the amount of PAH metabolites measured in the urine samples increased, the TAC concentration decreased. Previous research has also indicated that accelerating oxidative stress and reducing TAC affect the prevalence of lung cancer^33,34^.

Exposure to PAHs has been identified as one of many causes of adverse health effects. It has been suggested that this phenomenon could be due to oxidative damage. For example, some studies on PAHs have been conducted in vitro and in vivo. Therefore, MDA is a commonly used biomarker to assess oxidative stress. Based on our results, there was a significant correlation between MDA levels and all measured PAH metabolites except the control group. The results showed that MDA concentrations increased significantly with increasing concentrations of urinary PAH metabolites. Researchers found that urinary MDA levels were positively associated with hydroxy-PAH levels in a rural population from the North China Plain (p < 0.05)^35^. Epidemiological studies have also reported an association between exposure to PAHs and urinary concentrations of MDA^35^. For example, Bae et al.^36^ reported that urinary MDA concentrations increased significantly with increasing urinary 1-hydroxy pyrene concentrations. A similar result was discovered in this study. Cooking oil smoke is one of the main sources of PAHs. Kitchen workers are highly exposed because they do not wear respiratory protection^8^. Therefore, it can cause oxidative DNA damage and lipid peroxidation^12,37^. Urinary levels of 1-OHP and MDA often represent occupational exposure to PAHs and oxidative stress among kitchen workers and their neighbors^38,39^.

CRP is an inflammation marker that rises when the body is inflamed. In the present study, there was no significant relationship between PAH metabolites and CRP in any of the three study groups (Table 3). However, the results of one study demonstrated that levels of biomarkers for urinary PAHs are positively correlated with serum CRP levels^40^. Therefore, in the present study, it is possible that the effects of confounders did not show any association between serum CRP and PAH metabolites in the urine of the participants.

### Linear regression analysis

**Figure 3:**
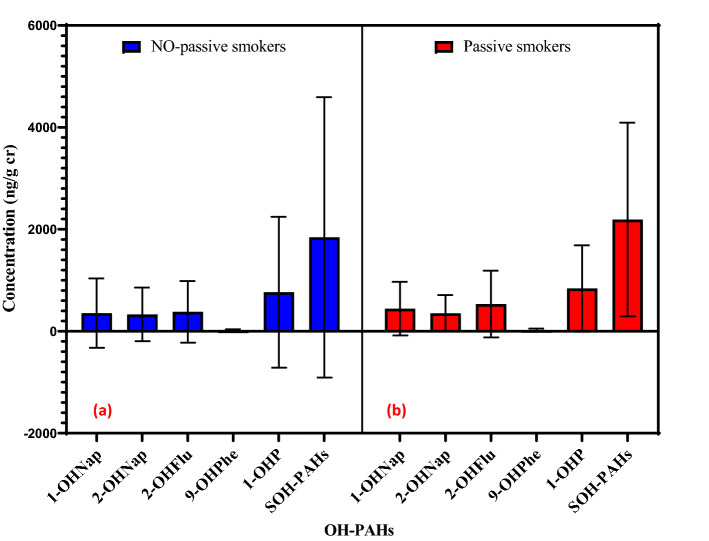
A comparison of mean PAH metabolites concentration (ng/g cr) in urine samples of (**a**) non-passive and (**b**) passive smokers.

The results of the regression analysis were used to examine the relationship between urine PAH metabolites levels and exposure variables such as subjects' activities, the cooking frequency at home and at work, age of the building, the use of the hood, the weekly consumption of food (meat, fish, grilled fruit and vegetables), passive smoking, body mass index, residence conditions in traffic, and etc. Only secondhand smoke had a significant relationship with the concentration of PAH metabolites in the urine of the studied participants (p < 0.05). 1-OHNap (β = 0.26 and p value = 0.013) and 1-OHP (β = 0.43 and p value = 0.01) were significant according to secondhand smoke in participants (Fig. 3). Therefore, this study considers secondhand tobacco smoke as a possible source of naphthalene and pyrene emissions. Additionally, several other studies have shown that cigarette smokers release significant concentrations of naphthalene into living spaces^41,42^.

### Non-carcinogenic and cumulative risk assessment

Most people believe that cooking and food processing techniques like smoking and drying are the main sources of PAH contamination. Cooking causes a variety of compounds, including PAHs, to be produced in food, depending on a number of factors, such as time, fuel used, distance from the heat source, drainage of fat, and type (grilling, frying, roasting). While the exact mechanisms of how PAHs are created are unknown, it is likely that there are several different ones, including the pyrolysis of meat at high temperatures and the pyrolysis of melted fat when it drips onto a heat source. As a result of eating more grilled and fast food, restaurant employees and people living nearby are exposed to PAH compounds through ingestion. In recent decades, health risk evaluation has been considered a valuable method for evaluating potential environmental risks^43^. We evaluated the risk of exposure to PAHs in the target subjects by only taking the dietary ingestion route into account because the diet is the primary source of PAHs exposure in non-smokers^25^.

**Figure 4:**
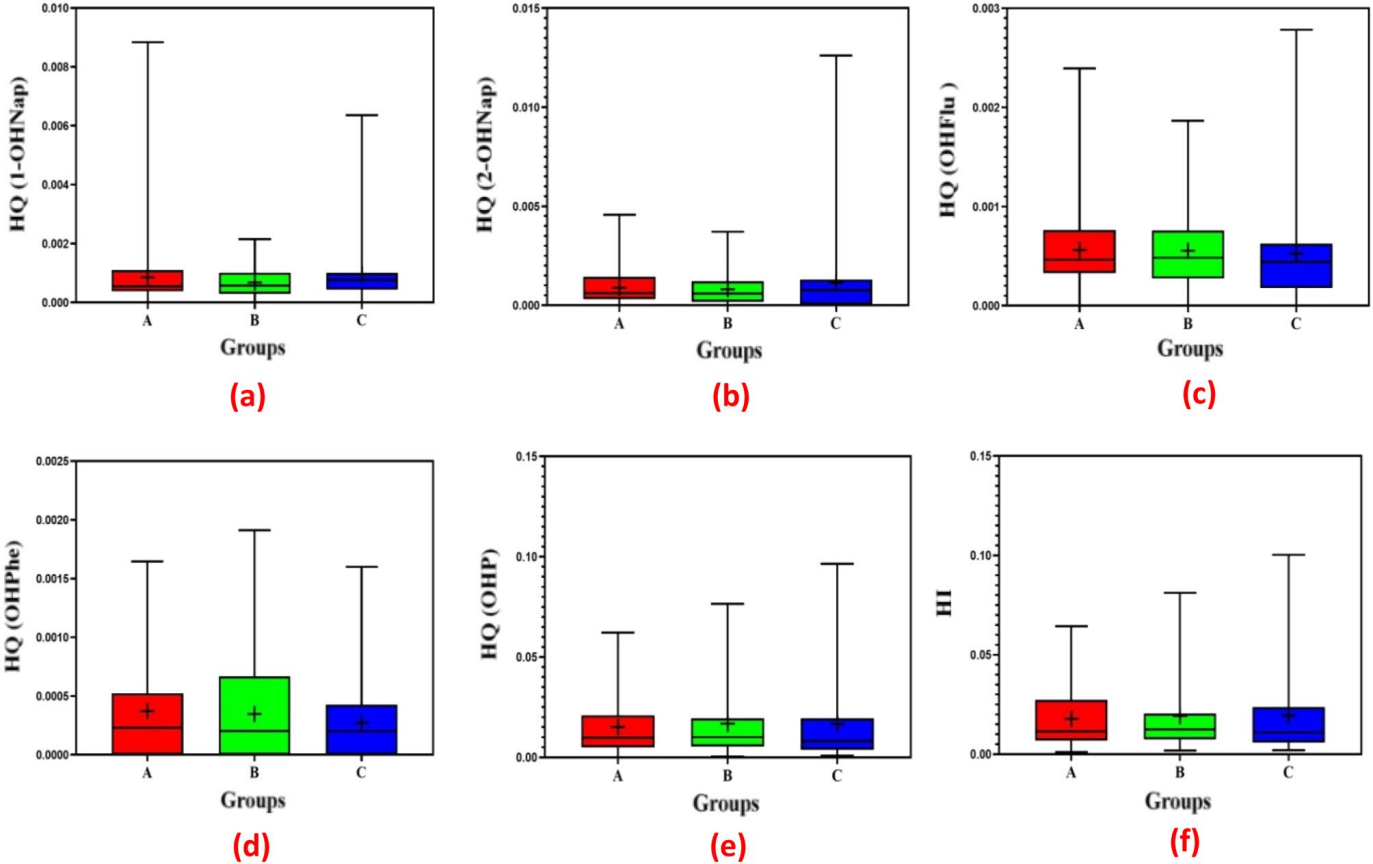
HQ_i_ for PAH metabolites ((**a**) 1-OHNap, (**b**) 2-OHNap, (**c**) 2-OHFlu, (**d**) 9-OHPhe, and (**e**) 1-OHP) and (**f**) HI_i_ in the studied groups (A: kitchen workers, B: people living near restaurants, and C: control group).

In the current research, EDI_i_, HQ_i_, and HI_i_ for 1-OHNap, 2-OHNap, 2-OHFlu, 9-OHPhe, and 1-OHP were estimated to investigate the potential health hazards of PAH metabolites. Table 4 shows the findings associated with EDI_i_. The results obtained in Table 4 were used to calculate HQ_i_, and HI_i_ based on Eqs. (2) and (3). The results related to HQ_i_ and HI_i_ are shown in Fig. 4. Based on the findings, HQ_i_ for PAH metabolites (1-OHNap, 2-OHNap, 2-OHFlu, 9-OHPhe, and 1-OHP) was less than one (HQ_i_ < 1). Furthermore, HI_i_ was less than one (HI_i_ < 1) in the studied groups, indicating low-risk negative health impacts on the target groups. Similarly, research conducted on Mexican children exposed to high levels of PAH_s_ found that the HQ_i_ was less than 1 in those children^44^. PAH_s_ exposure was not associated with any significant non-cancer health risks in another study conducted in Spain^25^. As a result of PAH_s_ exposure, Fernandez et al.^21^ found that Spanish women were not at significant risk of health. According to a study conducted in Mexico, women living in the studied communities had HQ_i_ greater than 1, which is associated with increased health risks^44^.

**Table 4:** EDI_i_ for PAHs metabolites measured in the studied groups.

PAHs metabolites	EDI_i_ (ng/kg-bw⋅day)		
	Kitchen workers	People living near restaurants	Control group
1-OHNap	17.11	13.56	19.11
2-OHNap	17.85	16.21	22.82
2-OHFlu	22.44	22.12	21.05
9-OHPhe	11.15	10.4	8.23
1-OHP	453.7	505.06	498.44

### Strength and limitations

Biomonitoring of restaurant workers and people living near restaurants was conducted for the first time in Shiraz, Iran. We examined the correlation between PAH metabolites and other measured clinical parameters to increase the reliability of the results of this study. Environmental PAHs can enter the body in several ways and cause a variety of side effects. The limitations of our study are mainly related to the small number of subjects in each of the three groups. Therefore, these results should be interpreted with caution so that other studies can support them. In future studies, larger sample sizes should be used for more detailed statistical analysis, especially for CRP, MDA, TAC, and RBC. It is suggested that future research examine how differences in diet and lifestyle affect emission sources.

## Conclusion

In this study, the levels of PAH metabolites in the urine and various clinical parameters in the blood of kitchen workers and residents near restaurants were examined. The average level of PAH metabolites in the urine of the kitchen staff was higher than that of the control group and residents of nearby restaurants. The mean concentration of 1-OHP was the highest among PAH metabolites. Additionally, MDA levels in kitchen workers and people living near restaurants were significantly correlated with PAH metabolites. The results of these studies demonstrated the negative linear correlation between TAC concentrations and PAH metabolite concentrations. There were no significant differences in the concentrations of clinical parameters between study groups. However, HI_i_ was observed to be less than 1 in all groups, indicating a low risk of adverse health effects for the target groups. In conclusion, identifying the sources of PAH emissions and reducing their production are key to reducing the harmful effects of PAHs on humans.

## Supplementary Information

Supplementary Information.

## Data Availability

The data that support the findings of this study are available on request from the corresponding author. The data are not publicly available due to restrictions, e.g., they information that could compromise the privacy of research participants.

## References

[1] Cachada A (2019). Urinary concentrations of monohydroxylated polycyclic aromatic hydrocarbons in adults from the US Population Assessment of Tobacco and Health (PATH) Study Wave 1 (2013–2014). Environ. Int..

[2] Zaj J, Gomó E, Szot W (2017). Urinary 1-hydroxypyrene in occupationally-exposed and non-exposed individuals in Silesia. Pol. Ann. Agric. Environ. Med..

[3] Wang Y (2019). Multivariate analysis for assessing sources, and potential risks of polycyclic aromatic hydrocarbons in Lisbon urban soils. Minerals.

[4] Tabatabaei Z, Hoseini M, Fararooei M, Shamsedini N, Baghapour MA (2022). Biomonitoring of BTEX in primary school children exposed to hookah smoke. Environ. Sci. Pollut. Res..

[5] Shahsavani S, Fararouei M, Soveid M, Hoseini M, Dehghani M (2021). The association between the urinary biomarkers of polycyclic aromatic hydrocarbons and risk of metabolic syndromes and blood cell levels in adults in a Middle Eastern area. J. Environ. Health Sci. Eng..

[6] Araujo JA, Nel AE (2009). Particulate matter and atherosclerosis: Role of particle size, composition and oxidative stress. Part. Fibre Toxicol..

[7] Khademi H, Khozeimeh F, Tavangar A, Amini S, Ghalayani P (2014). The Serum and salivary level of malondialdehyde, vitamins A, E, and C in patient with recurrent aphthous stomatitis. Adv. Biomed. Res..

[8] Pan CH, Chan CC, Huang YL, Wu KY (2008). Urinary 1-hydroxypyrene and malondialdehyde in male workers in Chinese restaurants. Occup. Environ. Med..

[9] Chen Y (2007). Gaseous and particulate polycyclic aromatic hydrocarbons (PAHs) emissions from commercial restaurants in Hong Kong. J. Environ. Monit..

[10] Jørgensen RB, Strandberg B, Sjaastad AK, Johansen A, Svendsen K (2015). Simulated restaurant cook exposure to emissions of PAHs, mutagenic aldehydes, and particles from frying bacon. J. Occup. Environ. Hyg..

[11] Ming-Tsang W, Lin P-C, Pan C-H, Peng C-Y (2019). Risk assessment of personal exposure to polycyclic aromatic hydrocarbons and aldehydes in three commercial cooking workplaces. Sci. Rep..

[12] Ciarrocca M (2014). Is urinary 1-hydroxypyrene a valid biomarker for exposure to air pollution in outdoor workers? A meta-analysis. J. Eposure Sci. Environ. Epidemiol..

[13] Singh A (2016). Heat and PAHs emissions in indoor kitchen air and its impact on kidney dysfunctions among kitchen workers in Lucknow, North India. PLoS One.

[14] Mo Z (2019). Characterization and health risk assessment of PM 2.5-bound polycyclic aromatic hydrocarbons in 5 urban cities of Zhejiang Province, China. Sci. Rep..

[15] Li CT, Lin YC, Lee WJ, Tsai PJ (2003). Emission of polycyclic aromatic hydrocarbons and their carcinogenic potencies from cooking sources to the urban atmosphere. Environ. Health Perspect..

[16] Shahsavani S, Dehghani M, Hoseini M, Fararouei M (2017). Biological monitoring of urinary 1-hydroxypyrene by PAHs exposure among primary school students in Shiraz, Iran. Int. Arch. Occup. Environ. Health.

[17] Shahsavani S, Dehghani M, Hoseini M, Fararoei M (2016). Health risk assessment of atmospheric paticulate-bound polycyclic aromatic hydrocarbons in Shiraz, Iran. J. Air Pollut. Health.

[18] Lewné M, Johannesson S, Strandberg B, Bigert C (2017). Exposure to particles, polycyclic aromatic hydrocarbons, and nitrogen dioxide in Swedish restaurant kitchen workers. Ann. Work Expos. Health.

[19] Chen JW, Wang SL, Hsieh DPH, Yang HH, Lee HL (2012). Carcinogenic potencies of polycyclic aromatic hydrocarbons for back-door neighbors of restaurants with cooking emissions. Sci. Total Environ..

[20] Kuczmarski MF, Kuczmarski RJ, Najjar M (2001). Effects of age on validity of self-reported height, weight, and body mass index: Findings from the third national health and nutrition examination survey, 1988–1994. J. Am. Diet. Assoc..

[21] Association WM (2009). Declaration of Helsinki. Ethical principles for medical research involving human subjects. J. Wissenschaft Ethik.

[22] García-García S, Matilla-González H, Peña J, Nogal Sánchez MD, Casas-Ferreira AM, Pérez Pavon JL (2022). Determination of hydroxy polycyclic aromatic hydrocarbons in human urine using automated microextraction by packed sorbent and gas chromatography-mass spectrometry. Int. J. Environ. Res. Public Health.

[23] Feldt T, Fobil JN, Wittsiepe J, Wilhelm M, Till H, Zoufaly A, Burchard G, Göen T (2014). High levels of PAH-metabolites in urine of e-waste recycling workers from Agbogbloshie, Ghana. Sci. Total Environ..

[24] Tabatabaei Z, Shamsedini N, Baghapour MA, Hoseini M (2022). Exposure assessment of children living in homes with hookah smoking parents to polycyclic aromatic hydrocarbons: Urinary level, exposure predictors, and risk assessment. Environ. Sci. Pollut. Res..

[25] Fernandez SF, Pardo O, Hernandez CS, Garlito B, Yusa V (2021). Children’s exposure to polycyclic aromatic hydrocarbons in the Valencian Region (Spain): Urinary levels, predictors of exposure and risk assessment. Environ. Int..

[26] Hemat H, Wittsiepe J, Wilhelm M, Müller J, Göen T (2012). High levels of 1-hydroxypyrene and hydroxyphenanthrenes in urine of children and adults from Afghanistan. J. Exposure Sci. Environ. Epidemiol..

[27] Singh A (2015). Assessing hazardous risks of indoor airborne polycyclic aromatic hydrocarbons in the kitchen and its association with lung functions and urinary PAH metabolites in kitchen workers. Clin. Chim. Acta.

[28] Siddique R (2021). Probing the impact of conventional oil frying on the formation of polycyclic aromatic hydrocarbons in rabbit meat. Food Sci. Nutr..

[29] Wang J (2011). elevated oxidative damage in kitchen workers in Chines restaurants. J. Occup. Health.

[30] Sawas A, Pentyala S (2004). Evaluation of lipid peroxidation in red blood cells by monitoring the uptake of sucrose and phenol red. J. Appl. Toxicol. Int. J..

[31] Fazeli, F. & Salimi, S. Correlation of seminal plasma total antioxidant capacity and malondialdehyde levels with sperm parameters in men with idiopathic infertility (2016).

[32] Eom SY (2013). Polycyclic aromatic hydrocarbon-induced oxidative stress, antioxidant capacity, and the risk of lung cancer: A pilot nested case-control study. Anticancer Res..

[33] Çalışkan-Can E (2008). Increased levels of 8-hydroxydeoxyguanosine and its relationship with lipid peroxidation and antioxidant vitamins in lung cancer. Clin. Chem. Lab. Med..

[34] Ito K (2012). Serum antioxidant capacity and oxidative injury to pulmonary DNA in never-smokers with primary lung cancer. Anticancer Res..

[35] Yang Q (2015). Polycyclic aromatic hydrocarbon (PAH) exposure and oxidative stress for a rural population from the North China Plain. Environ. Sci. Pollut. Res. Int..

[36] Bae S (2010). Exposures to particulate matter and polycyclic aromatic hydrocarbons and oxidative stress in schoolchildren. Environ. Health Perspect..

[37] Lai C-H (2013). Exposure to cooking oil fumes and oxidative damages: A longitudinal study in Chinese military cooks. J. Eposure Sci. Environ. Epidemiol..

[38] Ke Y (2016). Comparative study of oxidative stress biomarkers in urine of cooks exposed to three types of cooking-related particles. Toxicol. Lett..

[39] Pan CH, Chan CC, Wu KY (2008). Effects on Chinese restaurant workers of exposure to cooking oil fumes: A cautionary note on urinary 8-hydroxy-2-deoxyguanosine. Cancer Epidemiol. Biomark. Prev..

[40] Alshaarawy O, Zhu M, Ducatman A, Conway B, Andrew ME (2013). Polycyclic aromatic hydrocarbon biomarkers and serum markers of inflammation. A positive association that is more evident in men. Environ. Res..

[41] Batterman S (2012). Sources, concentrations, and risks of naphthalene in indoor and outdoor air. Indoor Air.

[42] Jia C, Batterman S (2010). A critical review of naphthalene sources and exposures relevant to indoor and outdoor air. Int. J. Environ. Res. Public Health.

[43] Franco SS, Nardocci AC, Günther WMR (2008). PAH biomarkers for human health risk assessment: A review of the state-of-the-art. Cad. Saude Publica.

[44] Pérez-Maldonado IN, Ochoa-Martínez ÁC, López-Ramírez ML, Varela-Silva JA (2018). Urinary levels of 1-hydroxypyrene and health risk assessment in children living in Mexican communities with a high risk of contamination by polycyclic aromatic hydrocarbons (PAHs). Int. J. Environ. Health Res..

