# Supplementary material for: Non-carcinogenic and cumulative risk assessment of exposure of kitchen workers in restaurants and local residents in the vicinity of polycyclic aromatic hydrocarbons
Source: Sci Rep. 2023 Apr 24;13:6649. doi: 10.1038/s41598-023-33193-0 (PMC10125965; doi:10.1038/s41598-023-33193-0)
Supplement: Supplementary file 1 — Supplementary Information. [file 41598_2023_33193_MOESM1_ESM.docx]

**Non-carcinogenic and cumulative risk assessment of exposure of kitchen workers in restaurants and local residents in the vicinity of polycyclic aromatic hydrocarbons**

Narges Shamsedini^a,b^, Mansooreh Dehghani^c,*^, Mohammad Reza Samaei^c^, Majid Nozari^d^, Shayan Bahrany^a^, Zeynab Tabatabaei^a^, Aboolfazl Azhdarpoor^c^, Mohammad Hoseini^c^, Mohammad Fararoei^c^, Sareh Roosta^e^

^a^ Department of Environmental Health Engineering, School of Health, Student Research Committee, Shiraz University of Medical Sciences, Shiraz, Iran.

^b^ Fars Water and Wastewater Company, Shiraz, Iran.

^c^ Research Center for Health Sciences, Institute of Health, Department of Environmental Health Engineering, School of Health, Shiraz University of Medical Sciences, Shiraz, Iran.

^d^ Department of Environmental Health Engineering, School of Public Health, Jiroft University of Medical Sciences, Jiroft, Iran.

^e^ MSc in Biostatistics, MA in General Psychology, Otolaryngology Research Center, Shiraz University of Medical Sciences, Shiraz, Iran.

**^*^Corresponding author:** Research Center for Health Sciences, Institute of Health, Department of Environmental Health Engineering, School of Health, Shiraz University of Medical Sciences, Shiraz, Iran. E–mail: mandehghani@yahoo.com, Tel: 98–9171184449; Postal Code: 7153675541.

**3.2. Distribution of PAH metabolites levels in urine**

Exposure to PAH_s_ is a significant concern because of its effects on human health. The mean levels of PAH metabolites in the urine samples of the study groups are displayed in Fig. 1 and other details are given in Table S1.

**Table S1.** **Statistical analysis of the urinary OH-PAHs in the studied groups**

|  | | Kitchen workers | | | | | People living near restaurants | | | Control group | | |
| --- | --- | --- | --- | --- | --- | --- | --- | --- | --- | --- | --- | --- |
|  | Median | | | (Min-max) | | Range | Median | (Min-max) | Range | Median | (Min-max) | Range |
| 1-OHNap | | | 154.92 | | (0-5229) | 5229 | 173.35 | (0-1847) | 1847 | 117.76 | (0-2545) | 2545 |
| 2-OHNap | | | 183.14 | | (0-3007) | 3007 | 197.12 | (0-2076) | 2076 | 104.3 | (0-2419) | 2419 |
| 2-OHFlu | | | 144.11 | | (0-3353) | 3353 | 246.88 | (0-2338) | 2338 | 93.12 | (0-3074) | 3074 |
| 9-OHPhe | | | 5.85 | | (0-153) | 153 | 7.28 | (0-109.99) | 109.99 | 2.67 | (0-51.67) | 51.67 |
| 1-OHP | | | 310 | | (0-9921) | 9921 | 400.99 | (0-5933) | 5933 | 190.81 | (25-7279) | 7253 |
